# Facilitators and barriers to COVID-19 testing in community and clinical settings: Lessons learned from Lesotho and Zambia

**DOI:** 10.1371/journal.pgph.0002430

**Published:** 2023-10-24

**Authors:** Musonda Simwinga, Palesa A. Mahlatsi, Masemote Molale, Gracious Witola, Isaac Mshanga, Bulemba Katende, Alain Amstutz, Klaus Reither, Kwame Shanaube, Masetsibi Motlomelo, Virginia Bond, Jennifer M. Belus

**Affiliations:** 1 Zambart, Lusaka, Zambia; 2 SolidarMed, Partnerships for Health, Maseru, Lesotho; 3 National University of Lesotho, Roma, Lesotho; 4 University of Basel, Basel, Switzerland; 5 CLEAR Methods Center, Division of Clinical Epidemiology, Department of Clinical Research, University Hospital Basel, Basel, Switzerland; 6 Swiss Tropical and Public Health Institute, Allschwil, Switzerland; 7 Department of Global Health and Development, Faculty of Public Health and Policy, London School of Hygiene and Tropical Medicine, London, United Kingdom; 8 Department of Clinical Research, Division of Clinical Epidemiology, University Hospital Basel, Basel, Switzerland; University of Washington, UNITED STATES

## Abstract

The sudden emergence of the coronavirus disease 2019 (COVID-19) had a devastating impact on health systems and population health globally. To combat the spread of COVID-19, countries enacted guidelines and safety measures, including testing, contact tracing, and quarantine. It was unclear the extent to which uptake of COVID-19 testing and other health initiatives would be accepted in countries with a history of dealing with widespread communicable disease transmission such as HIV or Tuberculosis. The objective of this study was to understand and compare the facilitators and barriers to COVID-19 testing at hospital sites in two rural communities in Lesotho and community spaces (referred to as hubs) in one urban community in Zambia during active phases of COVID-19 pandemic. Individual interviews and focus group discussions (FGDs) were held during March-October 2021 to explore facilitators and barriers to COVID-19 testing. FGDs with 105 community members and health care workers, and 16 individual interviews with key informants and four mystery shoppers were conducted across the two countries. In Zambia, four mystery shopper observations, and eight hub observations were also conducted. Individual country codebooks were developed and combined; thematic analyses were then conducted using the combined codebook. Findings were compared across the two countries, and most were consistent across the two countries. Two primary themes emerged that related to both barriers and facilitators: (1) structural conditions; (2) social implications and attitudes. The structural conditions that operated as barriers in both countries included public health isolation measures and misinformation. In Lesotho, the cost of tests was an additional barrier. The only structural facilitators were in Zambia where the community hubs were found to be accessible and convenient. The social implication barriers related to fear of isolation, stigma, and mental health implications because of quarantine, perceived pain of the test, and compromised privacy. Social facilitators that led to people testing included experiencing COVID-19 firsthand and knowing people who had died because of COVID-19. Across both countries, primary barriers and facilitators to COVID-19 related to structural conditions and social implications and attitudes. Public health measures can be at odds with social and economic realities; pandemic response should balance public health control and the socio-economic needs. Data from Zambia revealed that community-based settings have the potential to increase uptake of testing services. Community-based campaigns to normalize and reduce stigma for COVID-19 testing services are needed.

## Introduction

The identification and rapid spread of coronavirus disease 2019 (COVID-19), caused by severe acute respiratory syndrome coronavirus 2 (SARS-CoV-2) in early 2020, at a time when vaccines or treatments were unavailable, required governments and national health systems to rapidly shift their attention and health priorities. Many countries imposed strict lockdowns and restrictions on movement of people, along with a host of measures aimed at curbing the virus’ spread, including implementation of COVID-19 testing if one felt symptomatic or needed to cross provincial or national borders [1]. Despite the establishment of testing facilities, barriers persisted at all stages of the cascade, including screening, testing, isolation, and contact tracing and reducing the community-effectiveness of a trace-screen-test-isolate strategy [2].

As observed by Kavanagh and colleagues (2020), several factors have slowed down testing, ranging from high demand to insufficient staff and test kits, and logistical challenges such transporting specimen [3, 4] and inadequate diagnosis capacity [5]. Indeed, many African countries have been faced with several challenges to achieve massive testing, particularly at the beginning of the pandemic [6]. For instance, limited access and fake test kits were observed in Tanzania [7]. Moreover, other African nations like South Sudan, Ethiopia and Nigeria have also experienced trouble with testing capacity [8, 9].

To mitigate the challenges mentioned above, it is necessary to integrate accurate, rapid, and scalable point-of-care testing for the diagnosis of COVID-19 infection at the community level as a key tenet of disease control, both in terms of disease prevention and treatment [10]. This is particularly important in resource-constrained settings, where existing healthcare systems are overburdened. At the same time, many resource-constrained countries have experience with prior infectious disease outbreaks, some of which have become widespread, such as cholera in Zambia [11, 12] and HIV in Lesotho. Taken together, these experiences inform future pandemic preparedness and response. We conducted a comparative analysis in Lesotho and Zambia of the barriers and facilitators to COVID-19 testing. Specifically, data were taken from two different COVID-19 studies, one in each country, and this comparative qualitative study was conducted with the aim of exploring the barriers and facilitators to COVID-19 testing at the different testing sites and documenting community response to different COVID-19 testing options across hospital sites in two rural communities in Lesotho and community spaces (referred to as hubs) in one urban community in Zambia.

## Methods

### Nested study design

This study was nested within two country-specific COVID-19 studies with the aim of the current study being to implement and evaluate different COVID-19 testing strategies in resource-constrained settings. In Lesotho, a larger study focused on mitigation strategies for communities with COVID-19 (MistraL) project. As part of the initiative, the MistraL project established COVID-19 screening facilities at district hospitals in the Butha-Buthe and Mokhotlong districts in Lesotho. This nested qualitative study was conducted at these two sites. In Zambia, the TREATS-COVID-19 study aimed to measure and model the prevalence and spread of COVID-19 in one urban Zambian community [13]. The TREATS-COVID-19 study offered COVID-19 screening and testing, alongside HIV testing, TB screening, and other services at designated community points, which included tents located outside the health facilities, and mobile sites (hubs) located in the community. The criteria used in the identification of the spaces was developed by community representatives and included that places should be well known, visible, easily accessible, secure with access to basic water and sanitation facilities and electricity. The places which were identified included open places near schools, markets, churches, open grounds, bus stations, play parks and shopping areas. Tents were erected in these places early in the morning and dismantled in the evening for several days until testing saturation was reached in a particular area.

### Data collection and participant selection

Several qualitative data collection methods were used across the two countries to understand and explore barriers and facilitators to COVID-19 testing at the different testing sites. Specifically, these were hospital sites in rural Lesotho and community hubs in urban Zambia. There was a total of 125 participants (*n* = 48 in Lesotho, *n* = 77 in Zambia) who enrolled in this study and participated in different qualitative data collection methods, including individual interviews with key informants in Lesotho and mystery shoppers in Zambia, and focus group discussions (FGDs). In addition, 12 observations (eight general hub observations and four mystery shopper observations) were conducted in Zambia. Table 1 presents an overview of the enrolled participants, demographics, and qualitative data collection methods used.

**Table 1:** Methods and participant types.

Lesotho	**Methods**	**Participant Type**	**Participant Numbers/ observations**	**Gender**
	Individual interviews	Key informants, health workers, chiefs, community councilors, traditional doctors, spiritual healers, priests	16	7 women, 9 men
	Focus group discussions	Healthcare workers, Community members	32 (6 HCWs, 26 community members)	18 women, 14 men
Zambia	Observations	Hubs (community spaces). 8 general observations and 4 mystery shopper observations.	12 observations	Observations of general hub activities (not individual participants)
	Mystery shopper individual interviews	Community members	4	2 women, 2 men
	Focus group discussions	Community members–youths (18–24 years), men, women, community health committee, community COVID-19 committee. Included some who had/had not visited hubs.	73	41 women, 32 men

Participants from both countries were identified and selected using purposive sampling for their ability to provide rich contextual experiences regarding barriers and facilitators to COVID-19 testing. Participants selected had either had direct experience of COVID-19 or had stories to share. Thus, some had heard stories about the effect of COVID-19 from fellow community members, others had tested positive before, while others knew someone or a relative who tested positive and who consequently observed COVID-19 guidelines. Participants in Lesotho were selected based on location, their role in the community, and knowledge of COVID-19 happenings in the community. In Lesotho, village chiefs assisted in arranging for a public gathering (“pitso”) to sensitize and recruit potential participants. This was still allowable for priority research including for COVID-19 related research during the COVID-19 pandemic, but local health guidelines needed to be followed. In addition, community key informants were recruited individually through phone calls or during public mobilization. Health care workers were further identified with the help of hospital management. In Zambia, community members, known as mystery shoppers, were purposively selected with the help of Community Advisory Board (CAB) members based on their availability, willingness to be trained, willingness to conduct observations at the hubs, and having the intent to go to a hub even before they were asked to become mystery shoppers. The mystery shoppers were trained to conduct observations at hubs using a guide. Follow up individual interviews with the mystery shoppers were then conducted by a social scientist to capture and document a detailed account of their experience at the hubs. FGD participants were recruited based on exposure to hubs, Health Center Committee (HCC) representation, age, and gender. The HCC is a community-based organisation responsible for coordination of community health services. Two of the seven FGDs in Zambia were mixed gender groups 1) who had visited the hubs and 2) who had not visited the hubs. Participants were selected from areas of the community where the hubs were based, with the help of the CAB and the HCC. Public health measures were followed when conducting research activities, and these included social distancing and wearing face coverings.

### Ethical considerations

In Zambia, ethical approval for the study was obtained using an express mechanism set by the National Health Research Authority (NHRA), while in Lesotho ethical approval was given by the Lesotho Health Research Ethics Committee (ID 107–2020 Modify 01 and 02). Written informed consent was obtained from all participants, except for observations as this was not required and was part of the ethical approval. Participants also consented to have their interviews and FGDs audio recorded. Data collected for the study was anonymized during transcription. Pseudonyms were used in Lesotho, whereas participant identification numbers were used in Zambia. The Ministry of Health COVID-19 safety protocols and public health measures in each country were followed during data collection.

### Inclusivity in global research

Additional information regarding the ethical, cultural, and scientific considerations specific to inclusivity in global research is included in S1 Checklist.

### Data analysis methods

Interview and FGD audio recordings were transcribed verbatim. In Lesotho, they were translated and then transcribed into English, while in Zambia, those conducted in local languages were translated during transcription. Data analysis was informed by the framework and thematic analysis approaches [14] using emerging issues from the data (inductive) and from existing literature and the theoretical underpinning of the study (deductive), with the deductive analyses focusing on barriers and facilitators to testing uptake. Separate country codebooks were developed by social scientists from each country by reading and rereading up to six transcripts and identifying codes. The country codebooks and transcripts were shared across the two countries, and an inter-country codebook was developed that reflected similar themes, as well as themes unique for each country. Thematic analysis was then conducted by each country coding their transcripts using the inter-country codebook or themes.

## Results

Our results revealed two primary themes (1) structural conditions and (2) social implications and attitudes, which operated as both primary barriers and facilitators to COVID-19 testing options. Below, we describe these thematic areas as they relate to both barriers and facilitators; these data are also presented in Table 2.

**Table 2:** Thematic areas.

Broader themes	Barriers	Facilitators
Structural conditions	Contact tracing	Requirement for travel (proof of test)
	Isolation	Requirement by some institutions
	Lack of information and misinformation	Convenience/ space (Zambia only)
	Cost of testing (Lesotho only)	Free testing (Zambia only)
	Lack of incentives for testing	
Social attitudes and implications	Perceived pain from nasal swabs	Firsthand experience
	Fear of a positive result	Desire to confirm symptoms
	Fear of isolation	Positive experience, Convenience of space (Zambia only)
	Stigma	
	Contact tracing and isolation	

## Barriers

### Structural condition barriers

Public health isolation measures such as contact tracing and isolation, misinformation, the cost of tests, and lack of incentives to motivate people to test were structural condition barriers identified by community members.

The COVID-19 related public health measures in both countries required that all COVID-19 cases were registered (notified) and contact tracing conducted for all households with the cases. Furthermore, anyone testing positive could face isolation at home and, during early waves or for some at risk groups (for example, people with TB), isolation was required in health centers. Some community members feared the physical separation implications of these measures.

Contact tracing led to isolation of the people who tested positive for COVID-19. Isolation was a new phenomenon that was despised by most community members because it cut off social contact between family members and friends, both for those isolated within the household and more forcibly for those placed in isolation centers (when this regulation was in place). Those who feared to be isolated observed that their freedom of movement and related freedoms would be restricted. In the absence of a vaccine or cure, some people worried that getting a positive diagnosis and being isolated could even lead to death.

*“Some say once they test and the results come back positive*, *they end up having suicidal thoughts as they believe they’re already dead now that they are sick anyway”*. (Men, FGD, rural, Lesotho).

Misinformation about the origin of COVID-19 and doubts about its existence were strongest early in the pandemic but seemed to reduce over time until vaccines were introduced. Some people even believed that the two governments were using COVID-19 to get more funds from the west and China.

Early denial about the existence of COVID-19 translated into a low response to COVID-19 testing. However, a combination of experiencing COVID-19 related deaths and a gradual increase in reliable information from multiple sources encouraged informed uptake of COVID-19 testing.

“*As mentioned*, *we indicated that people believed that Corona is from animals*, *but this was when the disease was still only in China*, *there were a lot of myths then*. *Currently there are no myths*, *maybe there maybe a few in relation to the vaccine*, *they say after 2 years of getting vaccinated a person mutates and there are pictures circulating of people who have become albinos and wrinkly and they claim one will turn into a monkey as well”*. (Men, FGD, urban, Lesotho).

In Lesotho, people were required to pay for COVID-19 tests to obtain travel certificates, and community members complained about the cost of the tests. Participants reported that this discouraged many people from testing since they expected government to treat the COVID-19 situation as a pandemic emergency and to commit resources to ending it. This was particularly said to be common among individuals who work or have personal ties in South Africa and who therefore needed to cross the border.

“*The testing charge impacted me a lot*, *especially when I had to cross to South Afric*a, *it is expensive and I don’t understand why we had to pay*, *yet COVID-19 is a pandemic”*. *(Woman*, *52*, *KII*, *Lesotho)*.

Moreover, neither Zambia nor Lesotho provided incentives or reimbursement for people to test for COVID-19. This prompted community members to make comparisons with HIV testing projects or other COVID-19 studies, which compensated or reimbursed participants for their time. Some participants travelled considerable distances to testing sites, especially in Lesotho, and thus felt they should have been compensated.

### Social implication and attitude barriers

The social implications of having a COVID-19 infection (whether confirmed or suspected), fears of perceived pain of the test, and fears about the mental health consequences of isolation which could result from contact tracing activities were all barriers to COVID-19 testing. Many participants strongly feared the potential social implications of a positive test result, which would not only compromise their privacy if they ended up being isolated but could also open them up to possible harmful gossip from fellow community members. The latter could also happen because of contact tracing and Ministry of Health staff’s visit to households was said to automatically alert other community members of a potential COVID-19 case in the household visited. This highlighted stigma levels in the community.

“*People are scared of contact tracing*. *They are scared their neighbours will know and spread the word in the community”*. (Mixed gender and age, adults, FGD, Zambia.)

In addition to the social implications, there was a general perception among community members that the process of sample collection caused pain. This view persisted throughout the study but many people changed their opinion on the issue when they tested. In addition, community engagement was conducted in both countries to correct the impression that the discomfort experienced was not enough to cause pain.

“*I was nervous about coming here because of the COVID-19 test*. *I have heard a lot of people saying it is uncomfortable*. *My experience was okay*, *it wasn’t as bad as I thought it would be”*.

(Woman, 52, hub observation, Zambia).

Such sentiments shared by community members, who supposedly experienced the pain, prevented people from going to access COVID-19 testing. Some community members further argued that isolation could cause mental health problems, including suicidal thoughts. Those who mentioned this said that being isolated without the usual human contact, and the feeling that they could possibly die having been infected could trigger suicidal thoughts.

## Facilitators

### Structural condition facilitators

Community members were motivated to test for COVID-19 by multiple factors. COVID-19 test became a requirement for both local and international travel. One of these was the requirement to provide proof that one had tested and was negative to be allowed to travel. Even some workplaces and schools introduced periodic testing for their employees and learners to minimize transmission.

“*My workplace wanted to see my COVID-19 results that is the main reason I came here”*. (Man, 27, hub observation, Zambia).

In Zambia, participants said that the hubs were more accessible, closer to their homes and more convenient compared to government run health facilities, and this motivated them to test for COVID-19. Even the men liked the hubs as they could test whenever they wanted, and some said they did not have time to go to the health facility and wait to be attended to. Not only was COVID-19 testing free at the hubs, but results were also given quickly to the community members who tested. Consequently, community members compared service provision at the hubs and service provision at government run health facilities with some suggesting that hub staff were much more friendly compared to health facility staff.

“*I think most community members would mostly want to test for COVID-19 at the hub because it is not everyone who to go to the clinic to access the service*. *The goodness with the hub is that they are near people’s homes”*. (Woman, 22, mystery shopper, Zambia).

In Lesotho, testing was offered in the hospital settings, so no structural facilitators emerged with regard to the testing setting.

### Social and personal/ attitude level facilitators

Firsthand experience of the impact of COVID-19, such as seeing a person that one knows die from COVID-19 was a major motivation for some people to test. When the number of COVID-19 related deaths increased in each country, this convinced many people that the pandemic was not a hoax. Therefore, some people exhibiting COVID-19 related symptoms wanted to test to determine their COVID-19 status.

“*The fact that they had heard the COVID-19 fever is quite deadly*, *they wanted to get tested so that they can know if they have the virus or if it was just the normal flu”*. (Woman, 44, KII, Lesotho).

There were several sources of information about COVID-19 including radio, television, print media, influential individuals, and international organisations such as the World Health Organisation (WHO). Health facilities, healthcare workers, friends and relatives were the most trusted sources. As more accurate information became available, such as when lived experiences of having COVID-19 were relayed from health workers, close social networks as well as radio, and television, community members knowledge became more specific and bio-medically accurate about the different aspects of COVID-19 including symptoms, for instance the similarities between flu, TB, and COVID-19 symptoms.

## Discussion

This study explored the barriers and facilitators to COVID-19 testing in community settings in Lesotho and Zambia. Our findings suggest that barriers to COVID-19 testing fall under two broad categories: structural condition barriers (isolation, contact tracing, misinformation, cost of tests, lack of incentives) and social implications and attitude barriers (perceived pain, fear of a positive test, fear of isolation and stigma). Correspondingly, facilitators to COVID-19 testing also fall under the same broad categories; structural condition facilitators (proof of a negative test result as requirement for travel, requirement by institutions, convenience, free testing), and social implications and attitude facilitators (firsthand experience, convenience, desire to confirm symptoms, positive experience).

Overall, the barriers and facilitators were largely similar across the two countries, perhaps because of the similar socio-economic backgrounds of Lesotho and Zambia. The primary difference that emerged with respect to COVID-19 testing barriers was that tests related to travel were paid for out of pocket in Lesotho, while they were largely free in Zambia. The major difference in facilitators was that COVID-19 testing in Zambia was done in mobile community spaces called hubs. No structural facilitators emerged in Lesotho with regard to the setting of the testing, as testing was conducted in traditional hospital sites. Community members in Zambia had a positive experience of the hubs due to their convenient location, flexibility in the provision of COVID-19 testing, provision of free tests, and staff friendliness. Hubs were particularly convenient for men who rarely access conventional clinics/ health facilities. Research on men’s access to HIV testing services confirm that community based testing services, particularly hubs are popular with men [15]. Literature on acceptability of other health programmes, such as HIV testing and COVID-19 vaccines, confirm that geographical proximity of testing sites to the community increases uptake of health services [16, 17].

Fear, or lack thereof, moderated how individuals eventually navigated most of the barriers. The fear of testing at the personal/ attitudinal level was not only driven by the fear of perceived pain from nasal swabs, but also by the fear of a positive result. Fear at the personal level in turn fed into social barriers, such as fear of being isolated and being stigmatized by fellow community members. People feared that they would be put in isolation centers with little or no contact with their families if they tested positive. Others feared that contact tracing programmes, a structural barrier, would lead to them being identified by other community members as having COVID-19, which could lead to stigmatization (social implication). These findings highlight the importance of ensuring privacy and confidentiality, even during pandemics such as COVID-19 to reduce fear and to increase testing as well as improve adherence to public health measures. The COVID-19 testing related fears have been widely documented and have been attributed to reduced motivation for testing for COVID-19 [18]. Inadequate or lack of information about COVID-19 contributed to misinformation and doubts about the benefits of COVID-19 testing, thereby creating mistrust in government programmes and fed the cycle of fears. Such attitudinal related barriers have also been found to affect COVID-19 vaccination programmes contributing to mistrust of vaccine effectiveness and doubts about the benefits of vaccines [19].

Governments tried to reduce fear-based concerns to improve people’s attitudes towards COVID-19 testing through the implementation of Risk Communication and Community Engagement (RCCE) strategies. The strategies focus on the role of community engagement in addressing communication needs for different population groups [20]. In Zambia, as in other countries where RCCEs have been implemented, the groups identified and engaged included community leaders, religious leaders, learning institutions, traditional healers, teachers, and community health care workers [21]. However, the RCCEs need to be continually updated with new information [22] but the pandemic evolved at a fast pace such that taking new information/ material to remote areas was a challenge.

Community experience of COVID-19 at the beginning of the pandemic was negatively affected in part due to conspiracy theories. Such theories usually arise around significant events, and when information available is ambiguous to provide explanations as to why the events have happened, usually implicating powerful groups in society with supposed intent to manipulate others for their own interests. The COVID-19 is an example of such events [23, 24]. In both Lesotho and Zambia, some people said COVID-19 was a hoax and that their governments were taking advantage of the pandemic to access funding from donors. However, as information and number of deaths increased, perception of self-risk increased and the need to protect oneself and indeed others also increased. These findings are consistent with findings elsewhere that show that regarding COVID-19 prevention, people gradually began to care for the welfare of others as much as they did for their self-interest. This is borne from the realization that taking measures to protect oneself in turn protects the society at large [25]. A qualitative study on what motivates Americans to use or not use face coverings during the COVID-19 pandemic found that, among the reasons, people were demotivated by the misinformation (not sure whether face coverings are protective), and low perceived risk (the belief that they are not personally at risk). However, a high compliance with wearing face coverings was reported by people motivated by the desire to protect or respect others especially high-risk populations and family members, and a show of community responsibility (belief) that it is the right thing to do [26].

These findings must be considered in light of the study strengths and limitations. Strengths of the current study include a multi-country approach and involvement of multiple stakeholders in the two countries, which provided a broader context to the experience by community members. Another strength is that the study was conducted during the pandemic and therefore documented prospective lived experiences of community members and stakeholders. However, the pandemic developed and manifested in different ways across and within the two countries, such as times when the infection rates were high and more stringent measures needed to be in place. This meant that participants experience of the COVID-19 pandemic also varied, and thus some differences in responses may be a reflection of the underlying difference in pandemic trajectory. However, the consistency of responses overall assuages this concern. The methods used were not uniform across the two countries making a proper comparative analysis difficult. However, the study teams worked together to develop the tools and ensured that as much data that would allow cross country comparison was collected. The codebook was also jointly developed by the two countries.

In conclusion, our study found that both barriers and facilitators to COVID-19 testing in community and clinical settings exist. Knowledge of the barriers and facilitators, and how they manifest and affect people’s lives is needed to enhance compliance to COVID-19 public health measures. While the contribution of the public health measures to stemming the spread of the virus must be acknowledged, their effect on social and economic lives cannot be overlooked either, and achieving a balance between the two can improve compliance. Integration of COVID-19 testing in community and clinical settings can increase testing in overburdened health care systems in low and middle-income countries such as Lesotho and Zambia. However, such efforts must be accompanied with adequate community engagement and health promotion to mitigate against misinformation and to ensure that changes to the public health measures are informed by people’s lived experiences and the socio-economic reality of their lives.

## Supporting information

S1 Checklist Inclusivity in global research. (DOCX)

S1 File An inter-country code book. (DOCX)

S1 Dataset Lesotho dataset. (DOCX)

S2 Dataset Zambia data set. (DOCX)
