# Supplementary material for: Facilitators and barriers to COVID-19 testing in community and clinical settings: Lessons learned from Lesotho and Zambia
Source: PLOS Glob Public Health. 2023 Oct 24;3(10):e0002430. doi: 10.1371/journal.pgph.0002430 (PMC10597474; doi:10.1371/journal.pgph.0002430)
Supplement: S1 File — (DOCX) [file pgph.0002430.s002.docx]

**CODEBOOK**

**Manuscript title:** Facilitators and Barriers to COVID-19 Testing in Community and Clinical Settings: Lessons Learned from Lesotho and Zambia.

Codebook

Green = Zambia specific

Yellow Lesotho specific

No colour Both Zambi and Lesotho

|  | **Question/ Broad code** | **Data driven codes** | **Definitions of codes** |
| --- | --- | --- | --- |
|  | What are the reasons that encourage people to test for COVID-19? (Facilitators) | Possibility of traveling | People get tested if they needed to travel (cross the border)/ out of the country |
|  |  | Testing sites strategically located, visible and closer to homes | Hubs/ sites in the community are near to homes; did not have to walk long distances or get on a bus. |
|  |  | Free COVID testing and for other services |  |
|  |  | Offer of multiple services in addition to free COVID testing | HIV, COVID testing, TB screening offered in one tent; would not know what one is testing for |
|  |  | Trust/ confidentiality/ privacy | Assurance that study/ health staff will keep one’s information confidential, and the testing site/ hub offer privacy; can’t see what one is being tested for |
|  |  | Study/ health care staff attitudes | Welcoming; good interpersonal and communication skills; Friendly and Professional staff |
|  |  | Results given quickly, and conveniently |  |
|  |  | Positive experiences of friends or relatives | encouraged to test after hearing that their friend or relative tested without any pain or side effects. |
|  |  | Wanting to know COVID-19 status, having COVID-19 symptoms or when not feeling well | When ill, not feeling well, or have COVID-19 symptoms |
|  | | | |
|  | What are the reasons that discourage people to test for COVID-19? (Barriers) | Lack of enthusiasm/ lack of incentives | Reluctant to test; not offered anything after testing, no counselling nor medication; sent home to quarantine |
|  |  | Lack of incentives for the village health worker |  |
|  |  | Voluntary testing | Voluntary testing does not encourage testing |
|  |  | Fear of the unknown | Fear that one will likely start feeling sick when they know they positive; would stay health if didn’t know |
|  |  | Cost of testing | Cost of testing causes hesitation in decision making |
|  |  | Fear of nasal swab/ pain | Fear of the pain supposedly from the swab when inserted in nostril |
|  |  | Mixed feelings |  |
|  |  | Fear of a positive result/ Fear of being isolated/ restricted freedoms | Fear that one will be separated from family and isolated; staying indoors for 14 days can lead to depression |
|  |  | Fear of mental health issues | staying indoors for 14 days can lead to depression |
|  |  | Trust/ confidentiality/ privacy | Assurance that study/ health staff will keep one’s information confidential, and the testing site/ hub offer privacy |
|  |  | Study/ health care staff attitudes | Not welcoming; bad interpersonal and communication skills |
|  |  | Stigma | How community members are treated by relatives, friends, and others when they test positive |
|  |  | Fear of HIV test and test for other disease conditions | Community members afraid they would be diagnosed with other diseases such as TB |
|  |  | COVID-19 is a hoax |  |
|  |  | False feeling of protection by some vaccinated |  |
|  |  | Fear of COVID-19 vaccination | There was an ‘assumption’ that hubs were also vaccination sites |
|  |  | Fear of contact tracing | Fear that contact tracing would inadvertently reveal their COVID-19 positive status |
|  |  | Satanism | Fear that blood was being collected for Satanic ritual |
|  | | | |
|  | What are community members’ experience with testing? | Painful/ sore |  |
|  |  | Just irritating, not painful | Depends on the person doing the test |
