# Supplementary material for: Facilitators and barriers to COVID-19 testing in community and clinical settings: Lessons learned from Lesotho and Zambia
Source: PLOS Glob Public Health. 2023 Oct 24;3(10):e0002430. doi: 10.1371/journal.pgph.0002430 (PMC10597474; doi:10.1371/journal.pgph.0002430)
Supplement: S1 Dataset — (DOCX) [file pgph.0002430.s003.docx]

**LESOTHO Dataset**

| Domain/Interview guide question | Preliminary findings (codes and description) | Example quotes |
| --- | --- | --- |
| Contract tracing (Q8) | **-Fear of Stigmatization:** community members conceal their contacts due to fear of stigmatization  -**Lack of comprehension of contact tracing**: it is evident from the data that the concept of contract tracing should be unpacked first. Awareness and knowledge workshops are seriously needed. This is because the concept of contact tracing is ambiguous to the majority of the informants. This is despite efforts by the researchers to try to shed light.  **-Low testing statistics:** lack of testing from community members makes it hard to know the suspects.  -**Concealment of information:** withholding of information hinders the effectiveness of contact tracing. It is evident that some of the community members would rather treat themselves in secret which makes it hard for healthcare workers to trace their contacts.  -**Lack of cooperation:** key informants indicated that sometimes village health workers do not receive warm welcome from the community members such that if they happen to get a tip of from other community members it becomes a challenge to approach suspects.  -**Ineffectiveness:** Due to the lack of necessary PPE and medical equipment, contact tracing is not done effectively. The timely conduct of contact tracing – to pave the way for testing and isolation – is important to prevent further infections. Several delays in the process make rapid contact tracing challenging for COVID-19 since people are already infectious before their symptoms occur. Also, some people can transmit the virus even if they do not develop symptoms at all. | “People tend to distance themselves from those that are suspected to have COVID-19, they are still afraid to associate with them which leads to them feeling discriminated against or harassed”.(male,53, KII-Lesotho)  “Health workers have a good approach and work well with the public, it is the people who with-hold information from them”. (male,53, KII,Lesotho)  “Some people are afraid to interact with them while some say they do not have COVID-19 and continue to interact with them”. (Female, 54, KII,Lesotho)  “Health workers have a good approach and work well with the public, it is the people who with-hold information from them”. (male,53, KII,Lesotho)  “What I know is that families conceal when they have cases of COVID-19 and its hard getting involved in family stuff…”. (male, 42, KII,Lesotho)  “They do not have the information because we conceal it from them, and they are hesitant to approach us because we do not cooperate”. (male,53, KII;Lesotho).  “I am not sure, we have not experienced a high number of infections and most people in our region have not been tested like they have in other regions so we do not have such information hence I can’t say”. (male, 56, KII,Lesotho)  “Contact tracing is important especially for the other person to know that they might have been exposed to COVID-19 so that they can seek medical attention in time. It will also help to minimize the spread of this disease. However, other people do not like the idea. It feels like they are being harassed and being Covid-19 positive on its own is already hard for them”. (male,42, KII,Lesotho)  “In the case of contact tracing, we check up on people daily to monitor their progress, their temperature, we do not have the necessary PPE to keep on checking on someone. We are village healthcare workers that do not have the medical equipment.” KI-P07 |
| Testing for COVID and facilitators (Q15) | -**Knowing Status**: one informant stated that he did not experience any COVID-19 symptoms but found it compelling not only to adhere to the calls for testing but also to know his status. KI-P04 got tested but not because she had symptoms. But because test certificate was a necessary requirement for travelling abroad.  **- Exhibiting Symptoms/ Extremely sick:** Evidence show that community members and healthcare workers are still reluctant to get tested, they only test when they are extremely sick/exhibiting COVID-19 symptoms. It is also evident from the data that people are aware that it is important to test and most importantly when they experience Covid-related symptoms.  -**Loss of someone dear to COVID-19:** having someone dear who succumbed to COVID-19 has been identified as one of the influencers for people to find the need to test and stay safe  **-Possibility of traveling:** The community members expressed that people get tested if they needed to travel (cross the border)    -**Contacts of confirmed cases** | “I was just abiding by the set rules, also it is important to know my status”. (male,42, KII-Lesotho)  “In all honesty, as an individual and a healthcare worker, I attest to how difficult it is to get tested for COVID-19, so much that I decided to only get tested when I feel really sick”. (Female, 28, HCW,Lesotho)  “People go for testing only when they are seriously ill and feel obliged to test”.KII-P06  “…Some those who come to test you’ll find that they have lost a dear member of the family or relative etc, then they get that edge, and they also want to be safe, these are the ones that just come through with or without a reason”. (male,39, HCW,Lesotho) |
| Testing for COVID and barriers (Q15) | -**Cost of testing**: Although testing charge was not the reason why the minority of the informants did not test, it comes out that it is a barrier for many people.  **Uncertainty and fear of depression -** The community members are reluctant to get tested because after testing positive, they are not offered anything, no counselling nor medication, they are send straight home for quarantine which leads to depression.  The community have not accepted the idea of testing. It looks like they would rather not know their status at all.  Many factors have influenced people’s ability not to test, especially the community in question.  Fear of isolation and quarantine seems to top on the list. The community members are terrified of being locked up without their daily needs.There is also an assumption that undergoing isolation is likely to affect mental wellness.  **Fear of testing tool-** People are afraid of the nasal swab discourage testing.  **-Fear of stigma:** Sometimes community members are reluctant due to the stigma attached to COVID-19, this is mostly influenced by having the ‘COVID-19 vehicle’ having to pick up those who tested positive to bring them to care this apparently sparks gossips within the community, this have however changed with time as people became more knowledgeable and came to understand that everyone is likely to get infected by COVID-19 at some point in their life.  **- lack of trust in health system:** People have the fear that the testing tools are already infected  **-fear of loss of freedom:** Youths are mostly terrified of testing positive and having to be locked up at home without freedom of movement and falling behind with schoolwork for students.  **-Slow services:** Slow services caused by under-staffed discourage the community members to get tested. Healthcare workers indicated that even those who had already taken the initiative to come to the facility to get tested return back home after waiting for long hours. | “Yes it affects them a lot because when one test positive, having to quarantine for 14days becomes tricky as one would be wanting to go hustle for their kids, so this catches them off-guard and now they have to stay indoors, so it’s a challenge because it means there won’t be anything to eat while quarantining. So yeah it makes most not want to test. There was this other gent from around here, in the case of vaccinating, we are told that before one vaccinates, they must eat properly, so this guy vaccinated without eating and he had some challenging side effects as a result, it’s tough out here in the families”. (Male,52, FGD, Lesotho)  “As Basotho, {laughing} we always have many excuses for not testing. For example, they often say for instance say one is already sick and they’re living with this in their house without being aware and life goes on as normal, an external person may notice and make them aware that hey man, you are sick, all of a sudden now that they’re aware this will make them feel scared. Some say once they test and the results are positive, they end up having suicidal thoughts as they believe they’re already dead anyway now that they’re sick. So yeah, there’s still a lot of fear of testing as a result”. (male,37, FGD,Lesotho)  “I think another thing is that according to them, testing or not testing is pretty much the same as far as they’re concerned. This is because, even after the test and and the results are positive, there’s no medication or pills that one is given, you’re just told to go quarantine for 14days, so what’s the point of being indoors for 14days without undergoing any treatment or using anything to help you recover? So basically, even if they had not tested it would still be the same as one would still need/have to go out and hustle for food etc. So if perhaps after the tests and one is positive, they would be given something to use during the quarantine period, that would be a lot better”. (male,44, FGD,Lesotho)  ´’…Initially it was a major problem, they were really against it, one would literally fight you when trying to test them, they didn’t want to be swabbed in the nose, saying they heard that the swab is painful, that it actually reaches the brain when inserted and that they would be bleeding after the test’’. (male,22, HCW,Lesotho)  “Yeah a lot, because you’d find that in the villages, for instance a neighbour would test positive, we’d see the COVID-19 car popping through at their house and we start talking insinuating that the neighbour has COVID-19 and eventually this person is going to be stigmatised. Even us healthcare workers, when we go to people’s houses, they end up letting their dogs out on us because they don’t want us to come to their house so that they don’t get exposed, in the village, when people see a car with the COVID-19 sticker they start thinking whoever’s house it goes to has COVID-19. As you know Basotho are problematic, this stigmatisation then leads to the affected people not come through to the clinic for help due to the fear of the stigma, especially in the early days when COVID-19 emerged, but with time, their understanding became better and better, they’re no longer afraid of the stigma etc as they’re now aware that anyone can get infected by COVID-19”. (male,22, HCW,Lesotho)  “Some say those swabs already have COVID-19, meaning the test results are just going to come out positive anyway and tell them they have COVID-19 yet it isn’t the case”. (female,39, HCW,Lesotho)  “Yes, you’ll find that with some, for instance, we were testing at a school, as we were testing, that was also a way of conducting contact tracing as there would have been a kid who tested positive and we had to go to the school to test all the classmates, so we’d find that these kids aren’t being transparent and often hide their symptoms even though they know and can feel that they’re sick, they just don’t want to test. Some complain that they’re far behind with school work, so if they test and then have to go quarantine for another two weeks, this is going to set them back further on their school work because it’s not like the class will wait for him/her. So, such things make most people not comfortable and open up about their symptoms which in turn leads to them not testing and just disappearing. These were major challenges”. (male,22, HCW, Lesotho)  “it is the younger people who often get afraid, to an extent that they even get feelings of regret after testing, claiming it could have been better if they didn’t know anything and continued to live their lives normally”. (female,27, HCW,Lesotho)  “You would find that one even decided to leave yet they initially came to the facility to get tested”. HCW-08  “yes, it does. In a case where they must wait, they fail to understand why they have to wait for other patients to be consulted yet they arrived at the facility before them”. (Female,27, HCW, Lesotho) |
| Location of COVID testing (Q16 and Q19) (indicate if it was the KII or the community members) | **-Border gates,**  **-Hospital and Clinics**: The informants stated that they and the community members test at the local hospitals while other key Informants indicated the tested at the boarder gates. | “I just know that people that got tested because they were crossing to the other side (South Africa). One person was even sent back from the border gate because they had symptoms. And they came back home.” KI-P09 |
| Perception of community members about testing (Q18) | -**Under forced circumstances:** The informants expressed that people seemed to have found no reason, non-whatsoever to test. People do not test willingly but under compelling circumstances, e.g recommendation from school authority  **-Possibility of travel:** The informants expressed that people tested if they needed to travel (cross the border)  **-Experiencing covid symptoms:** although they wait until their situation is worse, people do have an understanding that it is necessary to test whenever they experience COVID-19-related symptoms | “People go for testing only when they are seriously ill and feel obliged to test”. (Female, 44, Lesotho)  “You will find that a person is compelled because their business requires.” (Male, 22, _HWC, Lesotho)  “I just know that people that got tested because they were crossing to the other side (South Africa). One person was even sent back from the border gate because they had symptoms. And they came back home.” KI-P09  “Myself included, we only got tested when we needed to, not when we suspected to have been infected”. (Male,37, FGD, Lesotho)  “In many cases people test when they suspect themselves. Also, when they travel from one place to another.”KI-P08 |
| Barriers and facilitators for COVID testing community members (Q20) | **-Mistrust:** The information shared by the informants suggests that people do not trust authorities and their leaders  **-Fear of Stigma and discrimination:** In the wake of the fear and uncertainty that emerge during a pandemic, stigma and discrimination quickly follow, exposing people to harassment and isolation. The stigma that arises during a pandemic can exacerbate existing inequalities, including those related to socioeconomic status and immigration status.  **Fear of Isolation and mental illness:** Stigma or stigmatization develops during outbreaks due to the human fear that arises from the anxiety about a disease of an unknown etiology, with the associated detrimental consequences on both individual and society levels especially when isolation and quarantine are involved.  **-Cost of testing:** It is clearly stated that the cost of testing is too high and thus discouraging testing.  **Discrimination,**  **-Individualism vs Collectivism**: In essence collectivism is the dominating factor because people feel as though they are abandoning/ discriminating their loved ones since “self-care” is viewed as “individualism”. They do not follow covid rules/protocol because of the fear of being discriminated if they test positive and their close friends/ family assist in breaking these rules to protect them. | “People thought they were targeted, information was taken to the village chiefs and councilors so they thought they were being monitored and would lose their jobs, they did not understand that it was important for people to stay at home to avoid crowding the workspaces”. (Male,53, KII, Lesotho).  “They say not knowing their COVID-19 status is better, so they would rather fall ill and recover without getting tested. The problem is testing positive and having to live with that information, and once they know it means people will also have that information and pay attention to them”. KI-P04  “People do not want to be admitted once diagnosed, they have expressed that they would die from depression if that ever happens”. KI-P04  “The testing charge impacted me a lot, especially when I had to cross to South Africa, it is expensive and I did not understand why we had to pay yet COVID-19 is a pandemic. For instance, our Bishop at church is originally from Bethlehem and they learnt that we as village health workers we will be conducting tests so they asked to get tested so that they can produce the results at the border to avoid the expensive tests’. KI-P02  “I knew that when many immigrants from South Africa travelled into Lesotho, people would hide those immigrants. This was because they were afraid that their people (i.e. friends and relatives) would test positive for COVID-19. It was evident that to some people the virus brings discrimination.” KI-P06  “That point I addressed that we will not abandon our people. They feel as though caring for themselves means abandoning their loved one. Rather, it is for the health safety of their loved one, so that they may continue to be with them and continue life with them. In a manner whereby people are caring for themselves.” KI-P06 |
