# Supplementary material for: Facilitators and barriers to COVID-19 testing in community and clinical settings: Lessons learned from Lesotho and Zambia
Source: PLOS Glob Public Health. 2023 Oct 24;3(10):e0002430. doi: 10.1371/journal.pgph.0002430 (PMC10597474; doi:10.1371/journal.pgph.0002430)
Supplement: S2 Dataset — (DOCX) [file pgph.0002430.s004.docx]

**ZAMBIA DATASET**

The data/ findings in this dataset are divided into three broad categories: barriers to COVID-19 testing,

facilitators to COVID-19 testing, and community members’ experience with testing.

Abbreviations:

1. CMTHBTTH = Community members that have been to the hub
2. CMTHNBTTH = Community members that have not been to the hub
3. CCT = Community COVID-19 Team

HCC = Health Centre Committee

**BARRIERS TO COVID-19 TESTING**

Lack of incentives

*“What do the hubs offer after being attended to? Nothing? Not even a K20?”* [Participant 2, FGD, CMTHNBTTH].

*“To motivate people to go and get tested, they should be given something like sugar, T-shirt, biscuits or milk”* [Participant 4, FGD, CMTHNBTTH].

*“The hubs not having K20 and T-shirts discouraged some people from visiting the hubs. Especially young men and women who used to go because of the T-shirt and K20”* [Participant 4, FGD, CMTHBTTH].

*“Yes, the T-shirts and K20 have affected the tents because when the K20s and T-shirts were being given, a lot of people where coming but now some people can’t come”* [Participant 9, FGD, Youths].

*“People used to get each other in groups to get tested, my friends would come and I would wait for them as I was already tested. Because of that, they were motivated. But now, people feel demotivated”* [Participant 3, FGD, Youths].

*“People are not scared of the location of the hubs, what encouraged them was the K20 and the T-shirts. But when they find no T-shirts or K20 they complain but they would have already been tested”* [Participant 1, FGD, Women].

*“That one of course, it affected the hubs. When it was changed, the change was not announced in the community when that study ended. After being attended to, people would ask about the T-shirt and K20. In the beginning others discouraged others over this. But at the moment they come, they have accepted”* [Participant 2, FGD, Men].

*“Whilst walking to the site 2women and one male asked if they would be given anything if they tested for COVID-19 at the hubs, but when they found out there were no T-shirts or money, they refused to get tested”* [Social Scientist, Hub observations].

*“My only request is that we want to be given T-shirts after testing”* [38years old man, Hubs].

*“People still want incentives such as K20 and T-shirts in order to be tested”* [Zambart staff].

Voluntary testing

Fear of the unknown

Fear of nasal swab/ pain (Misconceptions that the swab has COVID-19)

*“I think with the hubs there is one thing that is causing people not to go there, they fear the pain from the COVID test. There is need to sensitize people that you are no longer using the test method that reaches the brain”* [Participant 3, FGD, CMTHNBTTH].

*“I think why people are not coming is because they are scared of the COVID test. I think as a team, you need to organize a mobile team to sensitize the community that you are no longer using the testing method that reaches deep inside. This is another reason why people don’t visit the clinic even if they are sick”* [Participant 5, FGD, CMTHNBTTH].

*“I went during the first study were I was tested deep inside. Because of that, I am scared to test again”* [Participant 1, FGD, CMTHNBTTH].

*“I didn’t go because of the COVID-19 test. People are willing but they are scared of the COVID-19 test. They want a different method of testing”* [Participant 5, FGD, CMTHNBTTH].

*“I am scared of the COVID test, it’s very irritating. I was once tested and it was very painful. That is why we bypass the tents”* [Participant 2, FGD, CMTHNBTTH].

*“Some people say why should they be tested? when they are not sick. I know someone who had a flu and cough but they were scared of the COVID test. They said these days at the hospital they don’t test truthfully. Even when one does not have COVID-19, they will say they have it”* [Participant 7, FGD, CMTHNBTTH].

*“The concern I had was that people were saying that when one is tested for COVID-19, the pain in the nose, ear and head will continue being there. We need more sensitization to tell people that you no longer reach deep in the nose”* [Participant 6, FGD, CMTHNBTTH].

“Just like she has said, people were scared because they were told that after a COVID test, blood comes out and people get sick” [Participant 9, FGD, CMTHNBTTH].

*“I wanted to say that people are scared of the method used to test for COVID-19. This prevents them from going to the hubs. It would be better if another method could be used”* [Participant 6, FGD, CMTHBTTH].

*“The second tests they use don’t go deep in the nose, they don’t hurt. The first test they use go deep in the nose. They should just be using the second test”* [Participant 7, FGD, CMTHBTTH].

*“The process at the hubs is good, it is not long. People are scared of the vita PCR test, maybe COVID could be tested through blood”* [Participant 6, FGD, CMTHBTTH].

*“I did not access the services because I heard that the COVID-19 tests are painful”* [Participant 4, FGD, Youths].

*“Others have wrong information, they are lied to that the COVID-19 test is painful”* [Participant 8, FGD, Youths].

*“I think I am just scared of the nose swab”* [Participant 1, FGD, Youths].

“I am scared of the swab” [Participant 5, FGD, Youths].

“Some don’t go because of the wrong information that they have about testing for COVID-19; the pain of the swab and others are scared of contact tracing” [Participant 1, FGD, Youths].

*“What makes people not to test for COVID-19 at the clinic is because people say that at the clinic they don’t test well, it reaches the brain and blood comes out”* [Participant 8, FGD, Youths].

*“The other thing that discourages people is when people who have tested before say the COVID test is painful”* [Participant 7, FGD, Women].

“Others scare people when they say the entire tube enters the nose” [Participant 1, FGD, Men].

*“Others are ignorant that when I test for COVID-19 that is when I will have it. They think the COVID-19 test has COVID-19”* [Participant 3, FGD, Men].

*“A lot of people are scared to test for COVID-19 at the hubs because of the misconception that they have that the swab stick has the COVID-19 virus. Others were saying that most of the people who test for COVID-19 do not know how to test for COVID-19, they might make you deaf”* [22years old woman, mystery shopper].

*"I heard it is painful"* [Community member].

*“Despite being busy at the shop, I could have created time to come to the hubs, to be honest I was just scared. I was not scared to come to the hubs because of people seeing me but because of the tests”* [33years old woman, Hubs].

*“I asked him if it hurts because I heard they insert it very deep inside until blood comes out”* [63years old man, Hubs].

*“I was scared to get tested because people say you insert the test deep inside up to the forehead”* [30years old man, Hubs].

*“Today a staff passed by my house and told me the tests are not that painful and they don't reach deep inside like I was scared, that was what convinced me to come”* [69years old man, Hubs].

*“At first I was scared to get tested because I heard from people that the COVID-19 test pains”* [18years old man, Hubs].

*“When coming here I was nervous and scared at the same time because of the rumors I heard that the COVID-19 tests go up to the throat or brain”* [25years old woman, Hubs].

*“The rapid tests were introduced. Mass testing was done in the community. There was mandatory testing when one visited the health facility. People shunned coming to the facility due to fear of being tested”* [Community member, CCT FGD].

Fear of a positive result/ Fear of being isolated/ restricted freedoms

*“Some say, when they are found with COVID-19 at the clinic, they will be taken to the isolation Centre and people in the community will know”* [Participant 4, FGD, CMTHNBTTH].

*“The other thing that makes people scared is being taken to the COVID Centre, even when they have the symptoms. They are scared of being isolated and their relatives not visiting”* [Participant 8, FGD, Women].

*“For some, they are scared of being found with other diseases. They can know the signs of COVID-19 but can be scared to be found with it. They are scared about knowing about their health”* [Participant 7, FGD, Men].

*"My only fear is that, what if I am COVID-19 positive, I have a young child, where do I take her? And I have a shop to run"* [33years old woman, Hubs].

*“I was scared when coming to the tents, because it is cold and I move about in crowded places. I thought I might be found with COVID-19”* [44years old woman, Hubs].

*“People in the community shunned away from the health facility due to the fear of being found with COVID-19”* [Community member, CCT FGD].

Trust/ confidentiality/ privacy

*“Some people say why should they be tested? when they are not sick. I know someone who had a flu and cough but they were scared of the COVID test. They said these days at the hospital they don’t test truthfully. Even when one does not have COVID-19, they will say they have It”* [Participant 7, FGD, CMTHNBTTH].

*“There can be no privacy because the other client can hear what is being talked about”* [Participant 2, FGD, CMTHNBTTH].

*“At the clinic we feel shy because people will know what you are testing for because you will be in queues. Then we also find people we know there”* [Participant 6, FGD, CMTHBTTH].

*“Most people are scared to go to the clinic because of confidentiality issues that is why they go to Zambart, because they don’t know them. It would be best if you did everything. You find that someone ends up going to the clinic when they are very sick because of this”* [Participant 6, FGD, CMTHBTTH].

*“At times they are too open. They are close to the houses and some people who stay close to the hubs might not be free to go there”* [Participant 1, FGD, Youths].

*“Just to support number 1, there is not much privacy. Like some are placed near the road. So people fear going to the hub because they feel others will see them. I felt the same when I went there. I was scared that the people seeing me would think I had HIV”* [Participant 4, FGD, Youths].

*“There are those people who are scared to go to the clinic because of distance and no confidentiality. But when they see the hubs, they are encouraged”* [Participant 6, FGD, Youths].

*“When I am being tested for things like HIV, I like to be alone so that I can freely ask questions. Some people might not come to the hubs because of this, they need to be attended to one at a time”* [Participant 3, FGD, Youths].

*“Others fear being found with other diseases, especially those who live near the hubs. They prefer going to test somewhere far”* [Participant 8, FGD, Men].

*“The workers at the health facility make people scared to go to the health facility because they take information in the community. Issues of privacy, so people avoid going”* [Participant 6, FGD, Men].

*“I knew the client who came in, he is my neighbor. When I saw him, I asked the health workers about confidentiality and they said it was okay and that I should not worry. There was no confidentiality in the tent because I could hear what the other person was answering and they could also hear me”* [50years old man, mystery shopper].

*“I think the hub is not friendly to Community members because it is by the road side and anyone passing by can see when a person is being attended to. This might stop most community members from coming to the hub”* [50 years old man, mystery shopper].

*“Whilst in the hub, I could hear what the other person was being asked. Maybe some community members are scared to go to the hubs because they are in an open area and people can see who is inside”* [22years old woman, mystery shopper].

*“The only issue is that the market is too public and others would be scared to be tested here. In addition, the aspect of privacy is compromised as two people are attended to in the tents at once"* [46years old man, Hubs].

*“However, this place is too open, that is why some people are not coming to the hubs. I think what would be best is if you get a room here at the shops, there are some unoccupied shops that are hidden that you could use to test people from”* [63years old man, Hubs].

*“I feel the tent has no privacy because results are told when others are in the tent. I know one of the men I was with in the tent, he is dating my ex, and I could tell that he was listening to what I was saying”* [46years old man, Hubs].

Study/ health care staff attitudes

*“For some, they don’t go to the clinic maybe because they were not welcomed well”* [Participant 7, FGD, CMTHBTTH].

*“They fear going to the health Centre because of the queues and the workers are not friendly like at the hubs”* [50 years old man, mystery shopper].

Stigma

*“I think the location is good because here at the clinic people feel shy to go there because of stigma. Others with HIV get their medication from the XX hospital because it is further from their homes, living the clinic here. Others go to test for HIV far in XX where they are not known because of stigma”* [Participant 7, FGD, CMTHNBTTH].

*“At the Centre people are scared because one knows what one has gone to test for due to the rooms they enter. There is stigma there, but at the hubs, one can’t know what one has gone to test for. There is no stigma”* [Participant 7, FGD, Women].

*“Us Zambians like laughing at others when they are sick. This is why they think people who come to the tents are sick. People only like going to the clinic when they are really sick. The tents have opened our minds to knowing about our health, their job is perfect”* [Participant 7, FGD, Men].

*“Some people see good things in a bad manner. I had COVID-19 and I was taken to the isolation centre. I was found with it at 13hours and taken to the isolation centre at 19hours. I received so much stigma at the hospital, I didn’t like it. People stayed away from me. When I was at the centre none of my friends visited me, but if you want privacy, you can’t get well. So contact tracing is good”* [Participant 7, FGD, Men].

*“With regards to COVID-19 tests, I think it is better for people to be tested than testing themselves because I think those who would test positive for COVID-19 would hide the information and not go to health Centre due to self-stigma”* [50years old man, mystery shopper].

Fear of HIV test and test for other disease conditions

*“Others were scared to go to the hubs because of HIV testing, whilst others were not*” [Participant 3, FGD, CMTHBTTH].

*“Others were not scared and others till now are scared to go to the hubs. Some people are scared to know their HIV status”* [Participant 6, FGD, CMTHBTTH].

*“It is a two way thing, others could have been encouraged and others could have be discouraged. They might fear COVID and HIV tests. So it depends on the person”* [Participant 7, FGD, Women].

*“Us men we get sick of these diseases like STDs, we go to the pharmacy privately without telling our wives because we are scared. This is the reason why some men don’t come to the hubs. They don’t want to be found with diseases. They don’t want to only have one woman”* [Participant 1, FGD, Men].

*“In the community, people have accepted the hubs. They like the fact that they are near their homes, it is easy for them to go there. However, the mind-set of some is that of a long time ago of saying when I go there, they will find me with different diseases”* [Participant 6, FGD, Men].

*“For some, they are scared of being found with other diseases. They can know the signs of COVID-19 but can be scared to be found with it. They are scared about knowing about their health”* [Participant 7, FGD, Men].

“Others fear being found with other diseases, especially those who live near the hubs. They prefer going to test somewhere far” [Participant 8, FGD, Men].

*"I was scared when coming to the tent after I heard you will be testing for HIV but I decided to know my health status”* [19years old man, Hubs].

COVID-19 is a hoax

*“A lot of people did not believe that COVID-19 existed”* [Community member, HCC FGD].

*“They did not believe (in the existence of COVID-19) because they were not seeing a lot of sick people. Only the rich people could get sick”* [Community member, HCC FGD].

False feeling of protection by some vaccinated

*“Yes, that was the misconception that people had, that if you get vaccinated you can’t get COVID-19. That is not the case, the vaccine only reduces the severity of the virus”* [Community member, CCT workshop].

Fear of COVID-19 vaccination

*“It is true, some people fear because they think when they come to the hubs they will be vaccinated”* [Participant 3, FGD, CMTHNBTTH]

*“Yes, some people fear visiting the hubs under the impression that the hubs are vaccinating people”* [Participant 6, FGD, CMTHBTTH].

*“Yes, a lot think you give the vaccine, so they are scared to come to the hubs. Majority are scared”* [Participant 1, FGD, CMTHBTTH].

*“A lot fear going to the hubs because they have the impression that you give the vaccine. They are scared of the vaccine because of what they hear, that you die. From the time the vaccine came, I only know one person who has been vaccinated”* [Participant 4, FGD, Youths].

*“Others thought the tents were vaccinating people for COVID-19. They were scared their blood would clot but after the third wave, a lot started getting vaccinated to prevent themselves from COVID-19”* [Participant 6, FGD, Women].

*“I think it is both sides, others are scared to go to the hubs because they think that the vaccine is there and others go because they think that it is there”* [Participant 7, FGD, Women].

*“To say the truth, I thought they vaccinate at the hubs. I went there to get vaccinated and they told me they don’t. So this can cause those who are scared of being vaccinated from going to the hubs”* [Participant 7, FGD, Men].

Fear of contact tracing

*“People are scared of contact tracing. They are scared their neighbors will know and spread the word in the community”* [Participant 5, FGD, CMTHNBTTH].

*“Some people are scared of contact tracing. They are scared when one is found with COVID and people home are followed, everyone in the community will know”* [Participant 7, FGD, Youths].

*“Some don’t go because of the wrong information that they have about testing for COVID-19; the pain of the swab and others are scared of contact tracing”* [Participant 1, FGD, Youths].

*“Others are scared that the neighbors will know. But it depends with the kind of person to determine if they will go to the hubs or not”* [Participant 5, FGD, Women].

*“Yes, it does conflict with privacy and confidentiality because by following people, people wonder what they want at the house. But nothing can be done because that is the procedure”* [Participant 5, FGD, Men].

Satanism

*“The other thing that was scary was the drawing of blood from the arm. I heard it was a lot of blood and I wondered where they were taking it. I think this scared most of the people. The other thing was that the drawing of blood was happening during elections. People were scared that their blood was being taken for Satanism, for demonic political reasons”* [Participant 5, FGD, CMTHNBTTH].

*“Some fears that we hear about the hubs is that some people say the hubs get blood from people for Satanism”* [Participant 1, FGD, Men].

Queues/Distance/Waiting time (Health facilities)

*“Others don’t go because of delays there, you are kept in line waiting”* [Participant 3, FGD, CMTHNBTTH].

*“Others avoid the clinic because of staying in queues for a long time without being attended to”* [Participant 1, FGD, CMTHBTTH].

*“But at the clinic because you find a lot of people on a queue, some get scared that people might hear what is being talked about despite closing the door”* [Participant 3, FGD, Youths].

*“At the clinic you queue up for one test, then go and queue up for another test”* [Participant 1, FGD, Youths].

*“I think I support him, looking at the distance, some people were not going to the clinics”* [Participant 1, FGD, Youths].

*“They are discouraged because of the queues at the clinic”* [Participant 6, FGD, Youths].

*“They fear going to the health Centre because of the queues and the workers are not friendly like at the hubs”* [50 years old man, mystery shopper].

Delayed results (Health facility)

*“Some don’t go to the clinic because the results take time to come out”* [Participant 3, FGD, Women].

*“The good part is that the results are quick, what discourages people from testing is that the results take long. Especially this thing were they get your phone number and they say when we call you then you have COVID-19 and if we don’t call you then you don’t have it”* [Community member, CCT FGD].

**FACILITATORS TO COVID-19 TESTING**

Possibility of traveling/work requirement

*“My workplace wanted to see my COVID-19 results that is the main reason I came here. I work at XX mall”* [27years old man, Hubs].

*"We are coming here to be tested because the names for ECZ are out. They are requesting everyone to get a COVID-19 test and show proof of the test results. That is why we are here. Clinics don't have these tests, so we will get tested here and then the doctor at XX clinic will sign our forms"* [Community member, Hubs].

Testing sites strategically located, visible and closer to homes

*“The hubs are put in central places that attract people to enquire what is happening. So the locations are good because that is where people pass”* [Participant 5, FGD, CMTHNBTTH].

*“The hubs are good because they are close to people’s homes. People used to feel lazy walking to the clinics”* [Participant 1, FGD, CMTHBTTH].

*“The location site of the hubs is good because they are close to homes. Like number 5 said, they also had a mobile lab that tested people for TB and X-ray”* [Participant 2, FGD, CMTHBTTH].

*“The issue of the hubs being closer to homes also contributed to the increase”* [Participant 1, FGD, CMTHBTTH].

*“The tents are strategically located so that they are easily assessed by the community nearby”* [Participant 6, FGD, Youths].

*“The location of the hubs is good. Like for those who stay in XX, the hubs are closer to our homes”* [Participant 8, FGD, Youths].

*“From my side, I think the location of the hubs is okay. They prevent people from walking long distances because even if you go to the clinic, people will still see you. So the location is okay”* [Participant 3, FGD, Youths].

*“There are those people who are scared to go to the clinic because of distance and no confidentiality. But when they see the hubs, they are encouraged”* [Participant 6, FGD, Youths].

*“Yes, the hubs are visible. They are put in points that are within the reach of people and easy to access unlike at the health centres were when one goes there, they are referred to other health facilities that are specialized in the particular disease”* [Participant 6, FGD, Women].

*“I like the location of the hubs, it is good. At times they are put at churches, junctions and markets. They put them in important areas and it makes people curious to know what is happening”* [Participant 3, FGD, Men].

*“Just looking at them, the hubs attract people to know what is happening. They look good and attract people. They are found in busy area”* [Participant 7, FGD, Men].

*“I think the location is alright and clear. It makes people curious, others think it is empowerment when they see people going there, or think it is old age empowerment. They get curious and go there without knowing they are dealing with health”* [Participant 1, FGD, Men].

*“The way you have put these tents, you have put them well. They are along the road and it is easy to locate them. It is the same with the clinic, when someone goes there, you can’t know why they have gone there. Unless the person in the hub discloses the information”* [Participant 1, FGD, Men].

*“In the community, people have accepted the hubs. They like the fact that they are near their homes, it is easy for them to go there. However, the mind-set of some is that of a long time ago of saying when I go there, they will find me with different diseases”* [Participant 6, FGD, Men].

*“The hubs are good because they are near people’s homes, no queues, and the results come out fast. This reduces the burden from health facilities. People used to die in queues waiting for results. Even money here at the hubs you don’t charge”* [Participant 1, FGD, Men].

*“The hubs are near people’s homes, when people have any symptoms such as a cough or failure to breathe, all this is examined at the hub that is nearby”* [Participant 4, FGD, Men].

*“I think most community members would mostly want to test for COVID-19 at the hub because it is not everyone who can manage to go to the clinic to access this service. The goodness with the hubs is that they are near people's homes”* [22years old woman, mystery shopper].

*“I liked the location of the hub, it was strategically placed at a cross-roads. One road was going to XX tank, the other road leads to XX ward, the other leads to XX ward and the fourth is the one that leads to the entry to XX community settlement area. This joint is a busy joint where people criss-cross. People from XXi come with vegetables and charcoal for sale to XX, XX and XX. Hence, this joint is a good joint”* [70years old man, mystery shopper].

*“I think the tents are very convenient, rather than going to the clinic for these tests, community members can easily come here”* [30years old woman, Hubs].

*“In sample area 23, because our tents were at the market we had a lot of people coming from different areas in the community and very few from the actually sample area”* [Zambart staff].

*“I feel the tent can be easily accessed by community members, it is set up at the right place”* [46years old man, Hubs].

*“I feel the location of the tent is good because it can be easily spotted by whoever is passing by”* [63years old man, Hubs].

*“The tent has been placed at the right spot as this is the center”* [30years old man, Hubs].

*“I feel you have put the tent at the right place because this is the center, I came from the farms but still saw it. This place is good”* [32years old man, Hubs].

*“We have chosen these places because they are famous and are easily accessible by people. Others are trading places. These places are in all corners of the community, so all community members have a chance of being tested”* [Community member, CCT FGD].

Free COVID testing and for other services

*“When the Zambart staff was passing through the community, I really liked their approach and how well they explained. I was contemplating about going to the hospital because one has to pay for the X-ray, but at the hubs it is free”* [Participant 3, FGD, Women].

Offer of multiple services in addition to free COVID testing/ Assumption of other services being provided (vaccination)

*“It is a good idea in the sense that in one tent you get to learn about TB, HIV and COVID-19. You find everything at one place rather than going from place to place. So that is a very good thing”* [Participant 6, FGD, CMTHNBTTH].

*“Just to add on, this COVID-19, TB and HIV are deadly diseases. It is good that you are testing for these coupled with counselling. It would have been time wasting and expensive to test them at different points”* [Participant 3, FGD, CMTHNBTTH].

*“As for me I wanted to know my HIV and TB status because of being a widow, I can’t know what my husband had. After knowing I was well, I also decided to test for COVID-19”* [Participant 8, FGD, CMTHBTTH].

*“It is good because at times people can’t know one is testing for HIV. They might think it is COVID-19 or something else”* [Participant 8, FGD, Youths].

*“I think it is good because when you go there, they test you for not just one thing in one tent, unlike when one goes to the clinic. At the clinic you queue up for one test, then go and queue up for another test”* [Participant 1, FGD, Youths].

*“The additional services do encourage people to go to the hubs. But it depends with what one wants”* [Participant 8, FGD, Youths].

*“The process is good because all the tests are carried out, HIV, TB, COVID and everything. One gets to have a clear picture by doing everything”* [Participant 6, FGD, Youths].

*“The hubs have made things easy, people can now go for check-ups even when they are not sick. And information is reaching people easily. A long time ago, people would wait until they were very sick to the point of being carried in a wheelbarrow to go to the clinic”* [Participant 10, FGD, Women].

*“The other services contributed to people going to the tents to test for COVID-19”* [Participant 5, FGD, Women].

*“I think it is both sides, others are scared to go to the hubs because they think that the vaccine is there and others go because they think that it is there”* [Participant 7, FGD, Women].

*“The locations of the hubs are 100% good. Honestly speaking, this time unlike the past, people’s minds are open and willing to go to the tents. This is because this time there are a lot of diseases and people want to test”* [Participant 2, FGD, Men].

*“In the community many know how the hubs work. Like in my case, I came to have my chest checked, I was also tested for COVID-19 and TB. I went and told others. People say the tents are good. When I see the people I know, maybe out of 12 about 8 came”* [Participant 3, FGD, Men].

*“They are appropriate to be provided alongside COVID-19 testing because two services can be done at one point. You don’t need to go elsewhere to get the other test”* [Participant 4, FGD, Men].

*“It is good to be tested for more than one disease at once to know what is causing what”* [Participant 1, FGD, Men].

*“The provision of other services at the hub had an impact on people testing for COVID-19. Instead of going to the hospital they would go to the hubs that are easier and faster”* [Participant 6, FGD, Men].

*“To say the truth, I thought they vaccinate at the hubs. I went there to get vaccinated and they told me they don’t. So this can cause those who are scared of being vaccinated from going to the hubs”* [Participant 7, FGD, Men].

*“I asked someone I know who was standing by the tent and he told me the services you were offering. He told me you were testing for COVID-19, TB, HIV and the chest. That is how I decided to have myself tested”* [46years old man, Hubs].

*“I had no challenges locating the tent, I met one of your staff and she brought me here. They told me you were checking people's chest, testing for COVID-19 and HIV. I was interested and decided to come*” [48years old man, Hubs].

*“I saw the tent when I was passing by, I did not know that you were testing for COVID-19. I thought you were offering the COVID-19 vaccine that is what made me stop. I hear about the vaccine on the radio. I heard it protects people from COVID-19 that is why I want to be vaccinated”* [32years old man, Hubs].

Trust/ confidentiality/ privacy

*“I think the hubs are good because they have privacy. Unlike the clinic were people fear meeting people they know. The hubs have privacy and people can be free to say what they want to say”* [Participant 4, FGD, CMTHNBTTH].

*“To add on on what she said, I think there is privacy. I can go to the hubs and test for what I want without people knowing. But at the clinic, when people see me near the ART block, they will say that I am sick”* [Participant 5, FGD, CMTHNBTTH].

*“For us who came here at the clinical site, we would find one person in the tent. We liked this because there was privacy”* [Participant 4, FGD, CMTHBTTH].

*“What is nice about the hubs is that when you go there, one can’t see who is inside or what they are testing for. The other thing is that for one to leave here and go to the general hospital, those are expenses. People prefer the hubs”* [Participant 6, FGD, CMTHBTTH].

*“To add on what number 6 has said, the hubs have privacy because people don’t know what you are testing for. At the clinic, there is no privacy, once you go where people get ARVs from, they think you are sick”* [Participant 5, FGD, CMTHBTTH].

*“Actually I think there is privacy because you are just the two of you and you are free to express yourself. But at the clinic because you find a lot of people on a queue, some get scared that people might hear what is being talked about despite closing the door”* [Participant 3, FGD, Youths].

*“The tents have made things easy. Some men fear going to the clinics, hence, with the tents it is better as there are not a lot of people”* [Participant 9, FGD, Women].

*“Most people feel the hubs have a lot of privacy unlike the health facilities were there are a lot of people. Also at the health facilities people take a lot of time unlike the hubs”* [Participant 6, FGD, Women].

*“To add on, at the hubs one can’t know what one is testing for. But at the health Centre, one can know what service one is testing for because of the room they enter. This causes people not to test for certain diseases. The hubs have helped”* [Participant 1, FGD, Women].

*“These services are good because other people are scared at the clinic especially men. At the tent, one can be passing in the ground and they would go there without anyone knowing. Unlike at the clinic where there are a lot of people”* [Participant 10, FGD, Women].

*“The tents are better because there are not a lot of people. There is privacy”* [Participant 8, FGD, Women].

*“The hubs have privacy despite being in an open area. Everything that happens in the hubs remains there”* [Participant 3, FGD, Men].

*“I have been at the hub and I saw that there is privacy because those who attend to clients they attend to them one to one. If you are five, they will first attend to one and not all at once. What is discussed in the hub remains there”* [Participant 4, FGD, Men].

*“The hubs are convenient and fast. The hub services are appropriate as there is also privacy and it saves time”* [Participant 5, FGD, Men].

*“The hubs have very much privacy unlike the clinic where people gather in a queue were they can be seen by everyone. They all get interested to know why you are there but at the hub, there is secrecy”* [Participant 1, FGD, Men].

*“I think the hub is friendly to Community members because the welcome was good and the hub has privacy”* [40years old woman, mystery shopper].

*“According to the information that I had gathered from the surrounding immediate community members, people do willingly go to the hub. This indicates that the hub is friendly to the community members”* [70years old man, mystery shopper].

*“I was comfortable being in the tents and there was privacy”* [15years old lady, Hubs].

*“I think the tents have privacy because no one can hear what is being talked about”* [30years old woman, Hubs].

Study/ health care staff attitudes

*“My experience was good. I was welcomed well and asked questions. Before being tested, they made sure I understood why I was being tested”* [Participant 7, FGD, Youths].

*“My experience at the hub was good. I was welcomed well despite having a cough and flu. I was counselled well before being tested for HIV”* [Participant 3, FGD, Youths].

*“For me the reception was okay. Everything was explained to me before being tested. There was also privacy”* [Participant 6, FGD, Youths].

*“I have never heard anything bad about the hubs but only good. People in the community say the reception at the hubs is good and the counselling is good. Unlike at the health Centre were people say the reception is bad. People prefer the hubs”* [Participant 10, FGD, Women].

*“I think the hub is friendly to Community members because the staff are very welcoming”* [22years old woman, mystery shopper].

*“I think the hub is friendly to Community members because the welcome was good and the hub has privacy”* [40years old woman, mystery shopper].

*"I am happy to be here because the staff are very friendly and I feel free to express myself”* [30years old woman, Hubs].

Results given quickly, and conveniently (Hubs)

*“The process is okay because they are never told ‘no you should come back tomorrow’. I am sure the hub program can’t be rolled out. It should be an on-going program till a cure for COVID-19 is found”* [Participant 3, FGD, CMTHNBTTH].

*“A lot talk about the goodness of the hubs. They are happy with the COVID-19 tests and being given the results”* [Participant 6, FGD, Youths].

*“The process is just okay because one gets their COVID results there and then”* [Participant 7, FGD, Youths].

*“When testing for COVID-19 at the clinic, they say one should go get the results after a week as they don’t have rapid tests. But at the hubs, the results come out within 15minutes”* [Participant 3, FGD, Women].

*“The hubs have improved the number of people testing for COVID-19. At the clinic many would refuse because the process there is too long. You have different people to write your name and attend to you throughout the process. Whilst at the hubs, it is convenient and fast. Everything is done in one tent”* [Participant 7, FGD, Women].

*“At the health Centre they say we will call you, but at the hubs they test you and give you the results”* [Participant 5, FGD, Women].

*“The hubs are good because they are near people’s homes, no queues, and the results come out fast. This reduces the burden from health facilities. People used to die in queues waiting for results. Even money here at the hubs you don’t charge”* [Participant 1, FGD, Men].

*“The hubs are convenient and fast. The hub services are appropriate as there is also privacy and it saves time”* [Participant 5, FGD, Men].

*“Yes, very much because everything at the hubs is faster unlike at the clinic where one spends a lot of time at the clinic”* [Participant 6, FGD, Men].

*“Before coming here I went to XX clinic to test for COVID-19 but I was told my results wouldn't come out the very day. I decided to come here at XX clinic to see if my results would come out the very day because I cannot report for work without my results. Just as I was entering the clinic, I saw this tent. When I asked if they were testing for COVID-19 they agreed”* [27years old man, Hubs].

*“I went to general hospital yesterday and they told me that the people who test for COVID-19 were not working. My husband advised me to come here because you people attend to people quickly”* [45years old woman, Hubs].

*“The good part is that the results are quick, what discourages people from testing is that the results take long. Especially this thing were they get your phone number and they say when we call you then you have COVID-19 and if we don’t call you then you don’t have it”* [Community member, CCT FGD].

Positive experiences of friends or relatives

*“Yes, the hubs have increased the number of people testing for COVID-19. They are hearing the testimonies of us who have been tested at the hubs before”* [Participant 2, FGD, CMTHBTTH].

*“Yes, people ask me how I felt when I tested for COVID-19. When I say it was good, it encourages others to get tested at the hubs”* [Participant 8, FGD, CMTHBTTH].

*“Others go to get tested for COVID-19 when they see that their friend got tested without any side effects”* [Participant 1, FGD, CMTHBTTH].

*“Others don’t know how to read even if they see the banner. Others are curious when they see people going there, hence, they ask what is happening. Others are encouraged to go to the hubs by associates”* [Participant 5, FGD, Men].

*“My friend and I saw the tent when we were passing by. He got tested yesterday and hence, he advised me to get tested”* [30years old man, Hubs].

*“I had no challenge with locating the hub because my father told me and my siblings what was happening here. He was also directed here by his work mates when he had COVID-19”* [23years old man, Hubs].

Wanting to know COVID-19 status, health status, having COVID-19 symptoms or when not feeling well

*“But for this one, it is voluntary, people only go when they want to know their health status”* [Participant 6, FGD, CMTHNBTTH].

*“What influenced me to go to the hubs was because I wanted to know my health status. It does not mean everyone who goes to the hubs is sick”* [Participant 5, FGD, CMTHBTTH].

*“Others wanted to get tested to know their health status. It depends with the person”* [Participant 6, FGD, CMTHBTTH].

*“Then there were others who were happy about it. They wanted to know their health status because they didn’t know what they were sick of”* [Participant 1, FGD, CMTHBTTH].

*“We noticed we had a cough and flu. These symptoms are what caused us in our home to go to the hubs. But we were all negative”* [Participant 3, FGD, Youths].

*“Just like what number 3 has said, I had the symptoms”* [Participant 8, FGD, Youths].

*“Mainly people go to the tents because of COVID-19 tests. But they do want to know their health status, hence, they test for other diseases like; TB and HIV. Whilst some would only want one test”* [Participant 3, FGD, Youths].

*“Others go because they have COVID-19 symptoms”* [Participant 4, FGD, Youths].

*“I went to the hubs because I was not feeling well. I had a headache and my body was not feeling well”* [Participant 5, FGD, Women].

*“What motivated me to go to the tents at XX was because I was not feeling well, I had a cough. I was motivated when I was told they will get my blood and go to test it in Lusaka. I really wanted to get tested”* [Participant 7, FGD, Women].

*“I went to the hubs after the Zambart staff who were passing through the community explained the goodness that was found in testing. When I was not feeling well, I went to the tents”* [Participant 8, FGD, Women].

*“Others go to the clinic because they want to know if they have COVID-19”* [Participant 1, FGD, Women].

*“Others go to test for COVID-19 when they are not feeling well”* [Participant 5, FGD, Women].

*"I feel good coming to the hubs because I really need to know certain things concerning my body, especially my health status”* [46years old man, hubs].

*“Immediately I saw the tent I decided to come because I am free and love knowing about my health”* [35years old man, Hubs].

*"I was free when coming to the hub because I wanted to know my health status”* [38years old man, Hubs].

*“I am not scared to come to the tents because I want to know my health status as it is important”* [45years old man, Hubs].

*“I feel relieved visiting the hubs because I wanted to know my COVID-19 status”* [63years old man, Hubs].

*"I was happy to come here and test for COVID-19 because I wanted to know my health status”* [46years old man, Hubs].

*"I was happy to come here as I wanted to know my health status”* [48years old man, Hubs].

*"I was happy when coming to the tents because I wanted to know my health status, especially COVID-19 and HIV”* [20years old man, Hubs].

*"I was happy to go to the tent because I wanted to know how my health was. I was told by the staff that you are testing for COVID-19 so I decided to come”* [69years old woman, Hubs].

“I was happy to come here because I wanted to get tested, you can be moving about not knowing that you are sick” [63years old man, Hubs].

*“Initially I passed here earlier and I thought you were only testing for HIV, but when one of your staff called me when I passed by for the second time and told me you were testing for COVID-19 I decided to get tested”* [30years old man, Hubs].

*"I am happy to come here because I wanted to know my COVID-19 status”* [23years old man, Hubs].

Sensitization/MOH programs (Both Zambart staff and MOH staff)

*“We see a lot of adverts on TV from the Ministry of Health, sensitization adverts”* [Participant 3, FGD, CMTHNBTTH].

*“The hubs did a good job, those who were passing door to door were really explaining things to people. Even those who did not want to get tested were encouraged to go to the hubs. They were really trained well”* [Participant 1, FGD, Women].

*“Just here at XX clinic, there were staff who were sitting at the gate asking people to wear masks, wash their hands and go under the mango tree to test for COVID-19 before being attended to”* [Participant 5, FGD, CMTHNBTTH].

*“First of all, you sensitized people in the community door to door. You said you were bringing hubs in the community. So it was easy for everyone to go”* [Participant 7, FGD, CMTHBTTH].

*“The health have been sending people door to door to sensitize us about COVID-19”* [Participant 3, FGD, Youths].

*“The health go in churches to sensitize people about COVID-19 and inspect to see that the guidelines are followed”* [Participant 7, FGD, Youths].

*“They also put posters in clinics explaining more about COVID-19. This encourages people to get tested even if they didn’t intend to”* [Participant 4, FGD, Youths].

*“What made me go to the hubs was because of how the Zambart staff explained to me about COVID-19. People were scared that the COVID-19 test is painful. But the staff did a good job explaining”* [Participant 1, FGD, Women].

*“At the hubs, people are really explained to about the goodness of testing and everything. But at the health Centre, they just give you a book and they say go to the lab”* [Participant 1, FGD, Women].

*“They provide community sensitization on COVID-19. They have put measures that whoever visits the Centre is screened and tested for COVID-19 before they are offered any services”* [Participant 1, FGD, Women].

*“I have only seen community sensitization”* [Participant 7, FGD, Women].

*“Even mobile testing and vaccinating in the community”* [Participant 1, FGD, Women].

*“There was this time people from XX clinic were passing in the community saying there will be men’s screening. Very few men went as compared to women. But Zambart passes in the community sensitizing people, men are now more open to going to the tents to check their health”* [Participant 7, FGD, Men].

*“These people who were sensitizing about the hubs passed through my home and explained what was happening there. That is what made me go there”* [Participant 3, FGD, Men].

*“The health are sensitizing people about COVID-19 on the radio and people are going to test for COVID-19. Like here in XX, I heard the health moving about with a mega phone in the community talking about the vaccine and its goodness”* [Participant 7, FGD, Men].

*“The neighborhood health committee are sensitizing people in the community about COVID-19 and the vaccine”* [Participant 7, FGD, Men].

*“I did not have any challenges locating the tents because some people were passing through in the community and they told me where the tents will be located”* [45years old man, Hubs].

*“I did not have any challenges locating the tent, same people passed by the road side where I work and told me where the tent had been set up”* [46years old man, Hubs].

*“I had no challenges locating the tent, I met one of your staff and she brought me here. They told me you were checking people's chest, testing for COVID-19 and HIV. I was interested and decided to come*” [48years old man, Hubs].

*“I was visited by people who told me you were testing people for COVID-19”* [81years old man].

*“A Zambart staff found my friends and me in the road and brought us here”* [19years old man, Hubs].

*“The Zambart staff brought me to the tent together with my friends that is how I knew about the tents”* [20years old man, Hubs].

*"I met one of the staff in the road and he directed me here and told me you were testing people for COVID-19”* [63years old man, Hubs].

*“Today a staff passed by my house and told me the tests are not that painful and they don't reach deep inside like I was scared, that was what convinced me to come”* [69years old man, Hubs].

*“It was easy to locate the hub because I have a friend who works here and he encouraged me to come. He also explained what happens here. That is what gave me the courage to come”* [25years old woman, Hubs].

*“The rapid tests were introduced. Mass testing was done in the community. There was mandatory testing when one visited the health facility. People shunned coming to the facility due to fear of being tested”* [Community member, CCT FGD].

**COMMUNITY MEMBERS’ EXPERIENCE WITH TESTING**

Painful/ sore

*“The COVID-19 test I did in Chirundu in the nose was painful, but this one was not”* [35years old man, Hubs].

*“I did not test for COVID-19 because I still have that fear from my previous COVID-19 test, it was very painful and blood came out of my nose. But once I get rid of the fear I will be back"* [30years old woman, Hubs].

*“Both COVID-19 tests were a bit painful”* [46years old man, Hubs].

*“However, my tests were alright. They were a bit painful”* [18years old man, Hubs].

*“I was scared to come to the tents because people were saying that the COVID-19 tests were painful. Both COVID-19 tests were very painful the time I got tested here”* [15years old lady, Hubs].

*“Whilst waiting to be attended to I was so scared when I saw how the lady who was being tested acted, it looked painful. Both of my tests were a bit painful”* [30years old woman, Hubs].

*“Both tests are okay, they are a little bit painful”* [25years old woman, Hubs].

*“Some people used to complain about headaches, even crying after being tested using the other test. But the test you have showed us is good”* [Community member, HCC FGD].

Just irritating, not painful

*“Us who have tested before are the ones who discourage others from going because we tell them the tests are painful. When I tested, it was not painful. People from number 4’s place discourage her, they tell her it is painful”* [Participant 3, FGD, Youths].

*“When I was being tested, the staff kept on asking me how I was feeling, the test was just okay. It was not what I expected. People say it is too painful and they insert the swab deep it the nose. It was well for me”* [50 years old man, mystery shopper].

*“When I tested it was okay and the test was not painful”* [22years old woman, mystery shopper].

*“The staff then tested me for COVID-19 in the nose and in my mouth twice. I do not know why I was tested twice I was not given a reason. Both tests were not painful”* [40years old woman, mystery shopper].

*“The same swab stick was used on both my nostrils. When the swab was inserted into my nostrils I felt a tingly sensation that almost caused the formation of tears. But it was quickly done and all was restored to order”* [70years old man, mystery shopper].

*“The pain was moderate, I would advise others to test. The process is good, everything is okay with me”* [46years old man, Hubs].

*“I did the self-sample collection, the staff showed me how to do it, and it was not painful. I pricked myself in my nose. The staff also swabbed me in the mouth and nose, the test was just okay”* [35years old man, Hubs].

“When the staff tested me for COVID-19, the test was not painful” [38years old man, Hubs].

*"My tears are coming out because the tests were irritating my nose but they were not painful"* [20years old man, Hubs].

*“I had no problem with both tests as they do not hurt, people lie that the test hurts but it does not”* [63years old man, Hubs].

*“Both COVID-19 tests were okay and gentle”* [73years old man, Hubs].

*“Both COVID-19 tests were okay”* [48years old man, Hubs].

*“The COVID-19 tests were both okay”* [77years old woman, Hubs].

*“Both COVID-19 tests were not painful”* [81years old man].

*“Both COVID-19 tests were okay”* [19years old man, Hubs].

*“Both COVID-19 tests were not painful”* [20years old man, Hubs].

*“The COVID-19 tests were not painful”* [69years old woman, Hubs].

*“My experience with both COVID-19 tests was ok, they did not hurt"* [33years old woman, Hubs].

*“Both tests were okay, the only thing is that a bit of my tears came out, I don’t know why”* [27years old man, Hubs].

*“Both tests were good and I did not feel any pain, I only felt itchy”* [63years old man, Hubs].

*“Both COVID-19 tests were okay, they were not painful like the way people say they are”* [30years old man, Hubs].

*“Both tests were okay and were not painful”* [69years old man].

*“Both tests were okay and they were not painful”* [30years old man].

*“Both COVID-19 tests were not painful and I like everything about the tent”* [32years old man, Hubs].

*“Both COVID-19 tests were okay, they were not very painful”* [23years old man, Hubs].

*“The COVID-19 tests were okay, they were not painful but a little bit uncomfortable”* [25years old man, Hubs].

*"I was nervous about coming here because of the COVID-19 test. I have heard a lot of people saying it is uncomfortable. My experience was okay, it wasn't as bad as I thought it would be”* [25years old woman, Hubs].

*“Both tests were not painful, I was just scared because I thought I might be hurt. People say it hurts until blood comes out”* [44years old woman, Hubs].

*“Yes, I think the test is good. We used to hear rumors that it goes in deep, but they lied”* [Community member, CCT FGD].

Seeing others dying from/ having COVID-19

*“COVID-19 has reduced. Because after a lot of people died, a lot were going to the clinics and hubs to get tested”* [Participant 5, FGD, CMTHNBTTH].

*“But after a lot of people have died, people are willing to get tested”* [Participant 9, FGD, CMTHNBTTH].

*“Yes, it has increased the number because a lot of people were willing to be tested especially after a lot of people died in the past months. The hubs helped because they are near the communities”* [Participant 6, FGD, Youths].

*“Sometimes people go to the clinic to get tested when they see that someone they know has COVID-19. At the same time this can also cause someone to get discouraged to test”* [Participant 6, FGD, Women].

*“What prompted me to go and do the tests is that my young sister tested positive for COVID-19”* [Participant 6, FGD, Youths].

*“This same incidence also made people believe that there is COVID-19, hence, people are getting vaccinated and getting tested”* [Participant 7, FGD, Men].

*“After people died from the third wave, this caused people to be going to get tested”* [Participant 1, FGD, Men].

*“A drunk participant passed through the hub around 13:35hours asking to be tested for COVID-19 because he heard his uncle in Lusaka had died from it. However, he could not be attended to as he was not in his right state of mind”* [Social scientist, Hub observations].

*“I was happy to come to the tent to test for COVID-19 after hearing about the third wave that has just come. I heard about it on the TV”* [77years old woman, Hubs].

*“I decided to come because I saw a lot of people dying from COVID-19 on TV especially in India. I feared that maybe the same virus has come to Zambia”* [81years old man, Hubs].

*“We tested a lot of people during the June- July period and a lot of people came out COVID positive on the antigen test. The other thing is that most (-1s) people who were out of the sample area, when tested COVID positive would bring their relatives for testing the next day”* [Zambart Staff].

*"I came here because my husband was found with COVID-19 last week”* [45years old woman, Hubs].

*“I did not experience any challenges locating the hub. My father was found with COVID-19 here and hence, I came here with my siblings”* [18years old man, Hubs].

Including fear of going to the health facilities after health workers had COVID-19)

*“Some people were scared to go to the health facility after the health workers also contracted COVID-19. It happened at XX clinic”* [Participant 2, FGD, Men].
